# The Impact of Smartphone Addiction on Sleep Quality Among High School Students in Makkah, Saudi Arabia

**DOI:** 10.7759/cureus.40759

**Published:** 2023-06-21

**Authors:** Weam M Alahdal, Amani A Alsaedi, Aliyah S Garrni, Fawaz S Alharbi

**Affiliations:** 1 Preventive Medicine, Ministry of Health, Makkah, SAU; 2 Epidemiology and Biostatistics, Ministry of Health, Makkah, SAU; 3 Dental Hygiene, Ministry of Health, Makkah, SAU; 4 Nursing, Ministry of Health, Makkah, SAU

**Keywords:** makkah, high school students, sleep quality, addiction, smartphone

## Abstract

Background

Smartphones are Internet-accessible devices that everyone can use in any setting, and their popularity is growing. However, the pervasiveness of smartphone technology has raised concerns owing to its addictive effect among adolescents and its association with sleep quality and mental and physical health issues.

Objectives

This study aimed to determine the effect of smartphone addiction on sleep quality among secondary high schools in Makkah, Saudi Arabia.

Methods

The study was conducted on high school students in Makkah, Saudi Arabia, using an analytical cross-sectional design, from January 2023 to August 2023. The study used a multistage stratified random sampling technique to select participants. The data were collected from an online self-administered survey and analyzed using IBM SPSS Statistics for Windows (IBM Corp., Armonk, New York, United States).

Results

This study included 373 respondents. Among those, males represent two-thirds of the study population (66%), while females are 34%. The median age of the participants is 16 years old, and the interquartile range (IQR) is 6. Our results show that the median number of hours spent on mobile phones is six per day, and the IQR is 5. Furthermore, our findings reveal that the mean Smartphone Addiction Scale-Short Version (SAS-SV) score is 38.5 ± 10.8 out of 60, and the average global PSQI score is 6.63 ± 3.03 out of 21. The results show that the study participants have problems using smartphones longer than they intended, constantly checking them, and missing planned works due to smartphone use. There is a positive correlation between smartphone addiction and sleep quality (*r* = 0.261; *p* < 0.001), indicating that the respondents have worse sleep quality when smartphone addiction and the global PSQI increased.

Conclusion

Our study concluded that high school students in Makkah, Saudi Arabia, have high smartphone addiction. Moreover, high smartphone addiction is significantly associated with poor sleep quality. This study can help with the development of measures to improve better sleep quality among high school students.

## Introduction

Smartphones are technological devices that significantly impact people's daily lives, changing their habits and behaviors. The utilities and capabilities of these devices are increasing, and the foresight is that this tendency will grow in the following years. However, the problematic use of smartphones has increased dangerously [1]. Many studies demonstrate that smartphone addiction impairs sleep quality [2]. However, the number of studies on Saudi adolescents could be much higher. Indigent darkness in adolescents and young adults can result in long-term sleep problems, which may impact them in adulthood [3]. Young adults today have grown up with smartphones as an evident part of their daily lives [4]. The reason is that the usage of smartphones goes beyond routine calls, allowing one to enjoy games, online shopping, various social interaction activities, and administrative work anytime and anywhere, and has brought us a convenient life [5,6]. Smartphones have also been proven helpful for students as a potential tool to “learn anywhere” [7].

Despite the benefits, smartphone overdependence among adolescents is derived from their developmental characteristics and causes severe psychological, mental, and social problems [8,9]. The overuse of electronic gadgets has been significantly associated with sleeplessness as adolescents spend most of their evening time watching or using electronic media [10].

Sleep is a crucial predictor of general health and well-being, and getting a decent night’s sleep is critical for a person's consistently good physical and mental well-being [11]. The ideal amount of sleep that 13-18-year-old adolescents need is eight to 10 hours [12]. However, children in secondary schools only sleep for a short time [13]. A study in Brazil on 710 adolescents found that 58.06% of subjects who accessed the Internet between 19:00 and 21:00 slept poorly [14]. At present, adolescents aged 10-19 years are growing up immersed in digital media, which has both beneficial and harmful implications for their healthy development [15].

Considering that Saudi Arabia has the highest proportion of smartphone users in the Gulf Cooperation Council [9], this study aims to examine the effects of smartphone addiction on sleep quality among high school students.

Rationale

We are entering a new phenomenon of addiction that might be the most pervasive in human history. While we may not change our habits, at least we can be aware of them to recognize better the effect of smartphone addiction on sleep quality, especially in the most critical age [16]. The adolescence period is critical for an individual's future, and the rapid development during adolescence indicates that it may be a critical or sensitive period for future health and disease conditions [16]. This is the first research about the impact of smartphone addiction and sleep quality in Makkah, Saudi Arabia, among this age group.

## Materials and methods

Study design, setting, and sampling

This analytical cross-sectional study conducted among high school students in Makkah, Saudi Arabia, aimed to determine the impact of smartphone addiction on sleep quality. 

According to the National Ministry of Education, the population size of secondary schools in Makkah is 30,000 [17]. Using Epi Info™ from Centers for Disease Control and Prevention (CDC; Atlanta, Georgia) to calculate the sample size from the cross-sectional study, we found that the expected frequency is 58.7% [2], with a confidence level of 95% and an error percentage of 5%. The ideal sample size was 368. However, we included an additional 15% to account for the clustered design effect and missing data.

**Figure 1:**
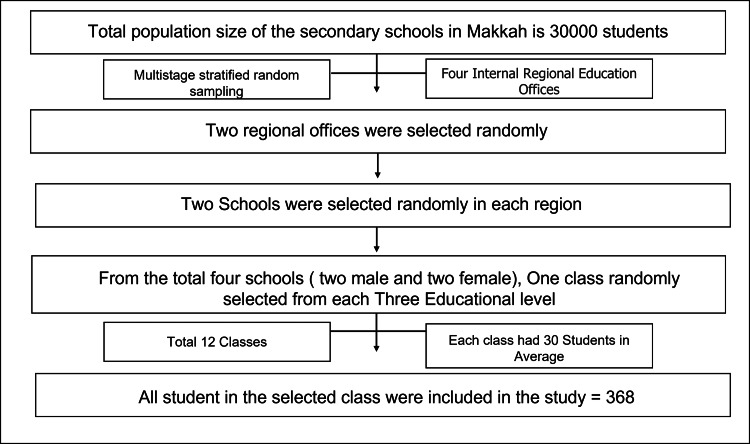
Flow chart of the sampling technique

A three-stage stratified random sampling technique was adopted. First stage: Makkah has four internal education offices; each education office includes several numbers of schools. Two regions were selected randomly using simple random sampling techniques. Second stage: Two secondary schools were randomly selected in each region using simple random sampling techniques. Third stage: In each school, one class was selected randomly in each education grade using simple random sampling techniques (Figure 1).

Data collection

The data collection used an online self-administrated form filled out by the students using the following tools: socio-demographic questions, including confounding factors that affect sleep quality, Smartphone Addiction Scale-Short Version (SAS-SV), and Pittsburgh Sleep Quality Index (PSQI).

SAS-SV: It is a valid and reliable Arabic tool [18] that contains 10 items rated on a dimensional scale (1 “strongly disagree” to 6 “strongly agree”). The total score ranges from 10 to 60, with the highest score being the maximum presence of “smartphone addiction” in the past year.

PSQI: It is a valid Arabic translation of the PSQI questionnaire [19]. It consists of 24 questions or items to be rated relating to the behaviors and experiences of the participants in the past month before the survey was conducted (20 items were rated from zero to three, while four were open-ended questions). It was not only developed to quantify sleep quality [20]; the PSQI can also be considered an accepted reference or gold standard for self-perceived sleep quality. In addition, it is the most widely used sleep health assessment tool in both clinical and nonclinical populations [21].

Inclusion Criteria: All Saudi and non-Saudi students enrolled at government and private secondary schools who have a smartphone and live in Makkah, Saudi Arabia, were included in the study.

Exclusion Criteria: Students with special needs, such as blindness, any participants with medical issues that affect sleep, and any participants under medications that can interfere with sleeping, such as first-generation antihistamines, were excluded from the study.

Ethical issues

Informed consent was obtained from each participant to know the purpose, benefits, and risks behind the study before they agreed to join. We considered anonymity and confidentiality; the participants can be in or out of the study at any time: no physical, social, psychological, or all other types of harm incurred. Approval was received from the Institutional Review Board (IRB) Opinion of the Ministry of Health, Makkah, Saudi Arabia, which was reviewed and discussed by the IRB Committee and was approved according to the International Conference on Harmonization for Good Clinical Practice (ICH GCP) guidelines with IRB Number H-02-K-076-0123-883.

Statistical analysis

The data were collected, coded, and cleaned. Data analysis was carried out using IBM SPSS Statistics for Windows, Version 23 (Released 2015; IBM Corp., Armonk, New York, United States). Descriptive statistics (i.e., percentage, mean, and standard deviation) were used to summarize the data. Moreover, statistical analysis using the Pearson correlation was applied with p-values <0.05 considered statistically significant.

## Results

Characteristics of the participants

A total of 373 subjects were included in this study. About two-thirds of the study participants (n = 246, 66%) are male, and the female respondents are 127 (34%). The median age of the participants is 16 years old, and the interquartile range (IQR) is 6. Our results revealed that the median number of hours spent on mobile phones is six per day, and the IQR is 5. Regarding the educational level of students, three high school academic levels were considered: first year, second year, and third year. We found that most of them are in the second year (n = 153, 41%), while 119 (31.9%) are in the first year and 101 (27.1%) are in the third year. Moreover, we found that most students are enrolled in government schools (n = 324, 86.9%), and only 43 (11.5%) are studying in private schools. In addition, the majority of students follow the general education path (81.5%), 23 (6.2%) follow the course system, 13 (3.5%) are in the standard first year, and 10 (2.7%) follow the computer science and engineering educational path. When we asked about the marital status of the respondents, we found that most of them are single (98.1%), and seven (1.9%) are married. Most of them live with their parents (85.5%), and 43 (11.5%) live with one of their parents. A total of 179 participants (48%) have a monthly family income of less than 5,000 Saudi riyal (SAR), 98 (26.3%) have a monthly family income of more than 10,000 SAR, and 96 (25.7%) have a monthly family income of 5,000-10,000 SAR. Only 65 students (17.4%) have a chronic disease. When we assessed the caffeine intake, we reported that 178 (47.7%) respondents consume caffeine less than six hours before bedtime. Only 105 (28.2%) perform sports activities regularly (Table 1).

**Table 1:** Sociodemographic data of the participants (n = 373) SD: standard deviation

Variable	Categories	N (%)
Age (in years)	Mean ± SD	15.8 ± 5.56
Average no. of hours/day spent on mobile phones	Mean ± SD	6.9 ± 4.45
Gender	Male	246 (66)
	Female	127 (34)
Educational level	1st year	119 (31.9)
	2nd year	153 (41)
	3rd year	101 (27.1)
School type	Government	324 (86.9)
	Private	43 (11.5)
	Other	6 (1.6)
Educational path	Common first year	13 (3.5)
	General	304 (81.5)
	Computer science and engineering	10 (2.7)
	Health and life	9 (2.4)
	Course system	23 (6.2)
	Literary	6 (1.6)
	Scientific	8 (2.1)
Marital status	Single	366 (98.1)
	Married	7 (1.9)
Living place	With parents	319 (85.5)
	With one of the parents	43 (11.5)
	Other	11 (2.9)
Monthly family income (in Saudi riyal (SAR))	<5,000	179 (48)
	5,000-10,000	96 (25.7)
	>10,000	98 (26.3)
Chronic disease	Yes	65 (17.4)
	No	308 (82.6)
Having caffeine less than six hours before bedtime	Yes	178 (47.7)
	No	195 (52.3)
Regular sports activity	Yes	105 (28.2)
	No	268 (71.8)

Smartphone addiction

Our findings show that the mean SAS-SV score is 38.5 ± 10.8 out of 60. SAS 9 ("Using my smartphone longer than I had intended") and SAS 8 ("Constantly checking my smartphone so as not to miss conversations between other people on Twitter or Facebook") of SAS-SV have higher means (i.e., M = 4.21, SD = 1.43; M = 4.19, SD = 1.52, respectively), followed by SAS 1 ("Missing planned work due to smartphone use") and SAS 4 ("Will not be able to stand not having a smartphone") (i.e., M = 4.05, SD = 1.48; M = 4.04, SD = 1.68, respectively). It indicates that the study participants have a problem using smartphones longer than they intended, constantly checking their smartphones, and missing planned works due to smartphone use (Table 2).

**Table 2:** Smartphone Addiction Scale Short Version (SAS-SV)

	Mean	Standard deviation
SAS 1	4.05	1.48
SAS 2	3.59	1.53
SAS 3	3.50	1.73
SAS 4	4.04	1.68
SAS 5	3.83	1.62
SAS 6	3.40	1.63
SAS 7	3.95	1.59
SAS 8	4.19	1.52
SAS 9	4.21	1.43
SAS 10	3.70	1.54
Total SAS score	38.46	10.78

Sleep quality

The average global PSQI score is 6.63 ± 3.03 out of 21. Based on the PSQI, the highest scores in sleep quality components were observed in component 2 (sleep disturbance; M = 1.36, SD = 0.89) and component 7 (sleep medication use; M = 1.36, SD = 0.70), followed by component 5 (sleep efficiency; M = 1.33, SD = 0.648) and component 3 (sleep latency; M = 1.11, SD = 1.08). The lowest scores were achieved by component 4 (daytime dysfunction due to sleepiness; M = 0.24, SD = 0.638) and component 6 (overall sleep quality; M = 0.29, SD = 0.681) (Table 3).

**Table 3:** Pittsburgh Sleep Quality Index (PSQI) component scores

Components	Mean	SD
C1 (Sleep duration)	0.95	0.82
C2 (Sleep disturbance)	1.36	0.89
C3 (Sleep latency)	1.11	1.08
C4 (Daytime dysfunction due to sleepiness)	0.24	0.638
C5 (Sleep efficiency)	1.33	0.648
C6 (Overall sleep quality)	0.29	0.681
C7 (Sleep medication use)	1.36	0.70
Global PSQI score	6.63	3.03

Factors associated with smartphone addiction (SAS-SV score) and sleep quality (PSQI score)

Our results show that the participants who spend a higher number of hours per day on mobile phones and those who have been taking caffeine less than six hours before bedtime are significantly associated with higher smartphone addiction (higher SAS-SV score; p-values < 0.001) and lower sleep quality (higher PSQI score; p-values < 0.001).

Moreover, several factors are significantly associated with higher smartphone addiction (higher SAS-SV score), including female respondents, students who live in other places rather than with their parents, and participants who did not perform a sports activity regularly (p-values = 0.042, 0.036, and 0.003, respectively).

In addition, the respondents who had monthly family income of less than 5,000 SAR were significantly associated with lower sleep quality (higher PSQI score) (p-value = 0.016). Other variables, such as age, educational level, school type, educational path, and marital status, did not show any significant association with smartphone addiction or sleep quality (p-value > 0.05) (Table 4).

**Table 4:** Factors associated with smartphone addiction (SAS-SV score) and sleep quality (PSQI score) *A higher SAS-SV score indicates higher smartphone addiction; a higher PSQI score indicates lower sleep quality.

Variable	Categories	SAS-SV score	P value	PSQI score	P value
Age (in years)	Spearman's rho	0.024	0.649	-0.002	0.970
Average no. of hours/day spent on mobile phones	Spearman's rho	0.331	<0.001	0.239	<0.001
		Mean ± SD		Mean ± SD	
Gender	Male	37.6 ± 10.9	0.042	5.9 ± 3.1	0.056
	Female	40.0 ± 10.3		6.5 ± 2.7	
Educational level	1st year	39.7 ± 10.6	0.211	5.8 ± 2.8	0.531
	2nd year	37.4 ± 10.6		6.3 ± 3.2	
	3rd year	38.5 ± 11.2		6.1 ± 2.7	
School type	Government	38.9 ± 10.6	0.097	6.1 ± 2.9	0.170
	Private	35.1 ± 2.0		5.8 ± 3.4	
	Others	38.0 ± 5.3		7.6 ± 0.9	
Educational path	Common first year	39.6 ± 10.4	0.231	6.7 ± 3.4	0.425
	General	38.7 ± 10.5		6.0 ± 2.9	
	Computer science and engineering	32.4 ± 9.7		6.7 ± 3.9	
	Health and life	36.3 ± 12.4		5.8 ± 3.3	
	Course system	38.4 ± 13.0		7.5 ± 3.3	
	Literature	43.7 ± 13.4		7.3 ± 2.4	
	Scientific	32.0 ± 9.4		5.6 1.1	
Marital status	Single	38.4 ± 10.7	0.834	6.1 ± 2.9	0.068
	Married	39.3 ± 14.2		4.0 ± 1.4	
Living place	With parents	37.9 ± 11.0	0.036	6.0 ± 2.9	0.252
	With one of the parents	41.9 ± 7.8		6.9 ± 3.4	
	Others	42.2 ± 12.2		5.5 ± 2.1	
Monthly family income (in Saudi riyal (SAR))	<5,000	38.8 ± 10.6	0.341	6.5 ± 2.8	0.016
	5,000-10,000	39.2 ± 10.5		5.8 ± 3.0	
	>10,000	37.1 ± 11.3		5.6 ± 3.1	
Chronic disease	Yes	40.2 ± 11.7	0.150	6.8 ± 3.2	0.058
	No	38.1 ± 10.6		6.0 ± 2.9	
Having caffeine less than six hours before bedtime	Yes	40.4 ± 10.2	0.001	6.8 ± 2.9	<0.001
	No	36.6 ± 11.0		5.5 ± 2.8	
Regular sports activity	Yes	35.8 ± 10.8	0.003	5.7 ± 2.8	0.134
	No	39.5 ± 10.6		6.2 ± 2.9	

 Predictors of sleep quality among the studied students

According to the multivariate regression analysis results, the participants who have a psychiatric disease, take caffeine less than six hours before bedtime, and have higher SAS-SV scores were significantly associated with poor sleep quality (p-values < 0.001). Other factors did not significantly affect sleep quality (p-value > 0.05) (Table 5).

**Table 5:** Predictors of sleep quality among the studied students SAS-SV: Smartphone Addiction Scale Short Version

Variable	B	Std. error	95% confidence interval for B		P-value
			Lower	Upper	
Age	-0.032	0.033	-0.096	0.032	0.331
Gender	0.214	0.334	-0.442	0.871	0.521
Educational level	0.121	0.230	-0.333	0.574	0.601
School type	0.085	0.386	-0.675	0.845	0.826
Educational path	0.162	0.144	-0.122	0.446	0.263
Marital status	-1.688	1.467	-4.575	1.199	0.251
Living place	0.089	0.384	-0.667	0.846	0.816
Monthly family income	-0.353	0.190	-0.726	0.021	0.064
Chronic disease	0.078	0.411	-0.730	0.886	0.850
Psychiatric disease	1.378	0.363	0.664	2.092	< 0.001
Having caffeine less than six hours before bedtime	1.055	0.309	0.448	1.663	0.001
Regular sports activity	0.021	0.341	-0.649	0.691	0.951
SAS-SV score	0.053	0.016	0.022	0.085	0.001
Average no. of hours per day spent on mobile phones	0.040	0.040	-0.037	0.118	0.308

Symptoms during sleep

Our findings show that more than half of the study population have a companion in the same room but not the same bed (n = 217, 58.2%). Moreover, most of the respondents (n = 170, 73.9%) stated that their roommate or bed partner did not observe them make loud snoring during the last month, and only 28 (12.2%) noticed snoring loudly less than once a week. The vast majority of students (n = 188, 81.7%) did not make long pauses between breaths while asleep. Only 41 (17.8%) were observed to experience leg twitching or jerking while they were sleeping less than once a week. In addition, most participants were not noted to have episodes of disorientation, confusion, or restlessness during sleep (above 70%). Twenty-eight (12.2%) had episodes of disorientation or confusion, and 27 (11.7%) experienced restlessness less than once a week during sleep (Table 6).

**Table 6:** Symptoms during sleep *There are missing data.

Variable	No bed partners or roommates	Partner/roommate in another room	Partner in the same room but not the same bed	Partner in the same bed
Do you have a bed partner or roommate?	131 (35.1)	12 (3.2)	217 (58.2)	13 (3.5)
If you have a roommate or bed partner, ask him/her how often in the past month you have had: (n = 230)	Not during the past month	Less than once a week	Once or twice a week	Three or more times a week
Loud snoring*	170 (73.9)	28 (12.2)	16 (7)	6 (2.6)
Long pauses between breaths while asleep*	188 (81.7)	21 (9.1)	4 (1.7)	4 (1.7)
Leg twitching or jerking while you sleep*	145 (63)	41 (17.8)	20 (8.7)	12 (5.2)
Episodes of disorientation or confusion during sleep*	172 (74.8)	28 (12.2)	13 (5.7)	6 (2.6)
Other restlessness while you sleep*	169 (73.5)	27 (11.7)	10 (4.3)	12 (5.2)

Correlation between smartphone addiction and sleep quality

The results show a positive correlation between smartphone addiction and sleep quality (r = 0.261, p < 0.001), indicating that the respondents had worse sleep quality when smartphone addiction increased and the global PSQI increased (Table 7).

**Table 7:** Pearson's correlation between smartphone addiction and sleep quality SAS-SV: Smartphone Addiction Scale-Short Version; PSQI: Pittsburgh Sleep Quality Index

	Global PSQI
SAS-SV	0.261
P-value	<0.001

## Discussion

The present study aimed to determine the effect of smartphone addiction on sleep quality among secondary school students in Makkah, Saudi Arabia. Internet addiction and smartphone addiction share many similarities. Based on how Internet addiction is defined, smartphone addiction is the excessive usage of cell phones to the point where it interferes with users’ daily life [22]. There needs to be more research on smartphone addiction, particularly among high school students.

The current study found that the median number of hours spent on mobile phone is six, and the IQR is 5 per day. The study participants have a problem using smartphones longer than they intended, constantly checking their smartphones, and missing planned work due to smartphone use. The results are similar to those in another study conducted in Saudi Arabia: 31.4% of the participants used a smartphone for one to five hours, and 50% (n = 156) used it for six to eight hours [23]. Moreover, our results revealed that the mean SAS-SV score was 38.5 ± 10.8 out of 60. It was relatively lower than that in another study conducted in India, which had a mean SAS score of 85.66 [24]. Another study in Saudi Arabia conducted among medical students showed that 54% of the participants had positive smartphone addiction [25]. Moreover, in our study, the lowest score was noted in the component of daytime dysfunction sleepiness. Daytime sleepiness score was also found to be low in a study conducted in Korea by Chung et al., which also demonstrated the link between daytime sleepiness to gender, alcohol consumption, and poor-self perceived health [26]. The disparities between these studies are most likely due to differences in evaluation systems and the sampled populations. For instance, in Saudi Arabian studies, the population was university students.

In this study, we found a significant positive correlation between smartphone addiction and sleep quality, indicating that the respondents had worse sleep quality when smartphone addiction and the global PSQI increased. It aligns with another study in India that revealed that those who used smartphones excessively had high global PSQI scores [25]. In addition, another study in Korea confirmed our findings and showed that higher S-Scale scores were associated with lower sleep quality [27]. The link between smartphone addiction and sleep quality was also reported in another parallel study in Turkey, in which smartphone addiction was associated with low sleep quality [28]. Moreover, prolonged and excessive smartphone use has been linked to musculoskeletal discomfort [29]. This is consistent with the findings mentioned in another Turkish study, which found an association between smartphone addiction and musculoskeletal pain [30]. Low mood was also found to be significantly associated with smartphone addiction (anxiety and depression) [4]. This finding also correlates with another study conducted by Hernandez et al., in which excessive use of smartphones was linked to the presence of depressive symptoms [31] and difficulty forming social connections [32]. Similar findings were reported in a congruent Australian study [33], which shows that smartphone addiction may impact sleep quality directly or indirectly. An earlier study found that evening exposure to electromagnetic fields affects physiological aspects of sleep and the melatonin cycle, likely by affecting the pineal gland. It may also impact the cerebral blood flow and brain electrical activity [34]. However, another contradictory study found that exposure to an environmental radiofrequency electromagnetic field did not interfere with the self-reported quality of sleep, but no evidence was found on the adverse effects of exposure to the radiofrequency electromagnetic field in our everyday environment on sleep quality [35]. Another study was conducted among undergraduate students in Makkah, Saudi Arabia, in 2021. A total of 545 undergraduate students, primarily females, aged ≤21 years old, showed that smartphone-addicted students were likely to have a lower GPA, poorer physical health, and severe mental illness than non-addicted students [36]. The differences in findings reported between the two studies could be attributed to different factors, including the studied samples and environmental factors.

Our study faces some limitations: The study employed a cross-sectional design, and hence the identified significant relationships between tested independent variables and the dependent variable (smartphone addiction) cannot be inferred as causal. In addition, the response rate from males was more than that from females, potentially because we collected the data of females during the final examinations. Furthermore, multiple factors can play an essential role in sleep quality, such as psychiatric and chronic diseases, which were not considered in this study [36,37].

## Conclusions

Our study concluded that high school students in Makkah, Saudi Arabia, have high smartphone addiction. Moreover, high smartphone addiction is significantly correlated with poor sleep quality. This study can help in the development of measures to improve better sleep quality among high school students.

Recommendations

It is vital to educate students about smartphone addiction and healthy sleep. In clinical applications with issues on poor sleep quality, students' use of smartphones and their addiction to them should be questioned. This study suggests that understanding smartphone addiction among adolescents requires further longitudinal studies to identify the causes of poor sleep quality and raise awareness in adolescents to change their negative behavioral patterns.

## Figures and Tables

**Figure 2:**
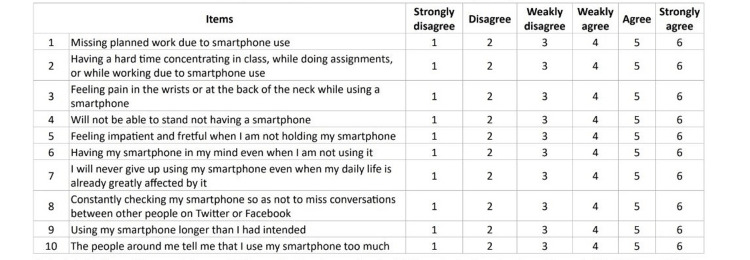
Smartphone Addiction Scale-Short Version (SAS-SV) Adapted from "The smartphone addiction scale: Development and validation of a short version for adolescents," by M. Kwon, D-J. Kim, H. Cho and S. Yang, 2013, PLoS ONE, 8(12), (https://doi.org/10.1371/journal.pone.0083558). Copyright 2013 by Kwon et al.

**Figure 3:**
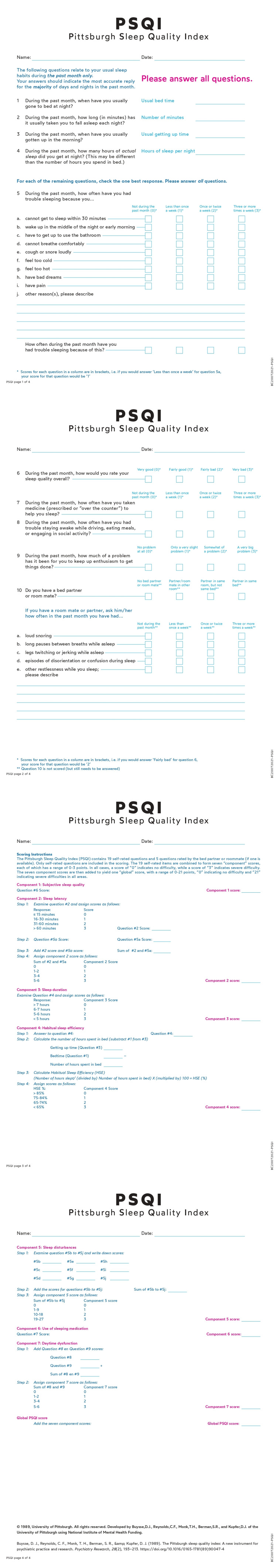
Pittsburgh Sleep Quality Index PSQI

## References

[1] Osorio-Molina C, Martos-Cabrera MB, Membrive-Jiménez MJ, Vargas-Roman K, Suleiman-Martos N, Ortega-Campos E, Gómez-Urquiza JL (2021). Smartphone addiction, risk factors and its adverse effects in nursing students: a systematic review and meta-analysis. Nurse Educ Today.

[2] Acikgoz A, Acikgoz B, Acikgoz O (2022). The effect of Internet addiction and smartphone addiction on sleep quality among Turkish adolescents. PeerJ.

[3] Bruce ES, Lunt L, McDonagh JE (2017). Sleep in adolescents and young adults. Clin Med (Lond).

[4] Tarokh L, Raffray T, Van Reen E, Carskadon MA (2010). Physiology of normal sleep in adolescents. Adolesc Med State Art Rev.

[5] Demirci K, Akgönül M, Akpinar A (2015). Relationship of smartphone use severity with sleep quality, depression, and anxiety in university students. J Behav Addict.

[6] Tegtmeier P (2018). A scoping review on smart mobile devices and physical strain. Work.

[7] Payne KB, Wharrad H, Watts K (2012). Smartphone and medical related app use among medical students and junior doctors in the United Kingdom (UK): a regional survey. BMC Med Inform Decis Mak.

[8] Stiglic N, Viner RM (2019). Effects of screentime on the health and well-being of children and adolescents: a systematic review of reviews. BMJ Open.

[9] Kuss DJ, Harkin L, Kanjo E, Billieux J (2018). Problematic smartphone use: investigating contemporary experiences using a convergent design. Int J Environ Res Public Health.

[10] Ettelt S, Nolte E, McKee M (2006). Evidence-based policy? The use of mobile phones in hospital. J Public Health (Oxf).

[11] Dutil C, Walsh JJ, Featherstone RB (2018). Influence of sleep on developing brain functions and structures in children and adolescents: A systematic review. Sleep Med Rev.

[12] National Sleep Foundation. (2016 (2016). National Sleep Foundation. How much sleep do we really need?. Sleep Res, 325.

[13] Echevarria P, Del-Ponte B, Tovo-Rodrigues L, Matijasevich A, Halal CS, Santos IS (2023). Screen use and sleep duration and quality at 15 years old: cohort study. Sleep Med X.

[14] Mesquita G, Reimão R (2010). Quality of sleep among university students: effects of nighttime computer and television use. Arq Neuropsiquiatr.

[15] Dworak M, Schierl T, Bruns T, Strüder HK (2007). Impact of singular excessive computer game and television exposure on sleep patterns and memory performance of school-aged children. Pediatrics.

[16] Viner RM, Ross D, Hardy R (2015). Life course epidemiology: recognising the importance of adolescence. J Epidemiol Community Health.

[17] (2021). Ministry of Education. http://moe.gov.sa.

[18] Sfendla A, Laita M, Nejjar B, Souirti Z, Touhami AA, Senhaji M (2018). Reliability of the Arabic Smartphone Addiction Scale and Smartphone Addiction Scale-Short Version in two different Moroccan samples. Cyberpsychol Behav Soc Netw.

[19] Suleiman KH, Yates BC, Berger AM, Pozehl B, Meza J (2010). Translating the Pittsburgh Sleep Quality Index into Arabic. West J Nurs Res.

[20] Buysse DJ, Reynolds CF 3rd, Monk TH, Berman SR, Kupfer DJ (1989). The Pittsburgh Sleep Quality Index: a new instrument for psychiatric practice and research. Psychiatry Res.

[21] Mollayeva T, Thurairajah P, Burton K, Mollayeva S, Shapiro CM, Colantonio A (2016). The Pittsburgh sleep quality index as a screening tool for sleep dysfunction in clinical and non-clinical samples: A systematic review and meta-analysis. Sleep Med Rev.

[22] Kim H (2013). Exercise rehabilitation for smartphone addiction. J Exerc Rehabil.

[23] Algarni SA, Aljohani AS (2021). Effect of smartphone addiction on sleep quality among medical students at Taibah University, Medina, Saudi Arabia. Med Sci.

[24] Soni R, Upadhyay R, Jain M (2017). Prevalence of smart phone addiction, sleep quality and associated behaviour problems in adolescents. International Journal of Research in Medical Sciences.

[25] Alsumairi NA, Alwagdani HA, Aloufi AO (2022). Impact of smartphone overuse on sleep quality among medical students in Taif, Saudi Arabia. International Journal of Medicine in Developing Countries.

[26] Chung JE, Choi SA, Kim KT (2018). Smartphone addiction risk and daytime sleepiness in Korean adolescents. J Paediatr Child Health.

[27] Chi S, Ko MS, Lee JH, Yi HS, Lee MS (2022). Smartphone usage and sleep quality in Korean middle school students during the COVID-19 pandemic. Psychiatry Investig.

[28] Kaya F, Bostanci Daştan N, Durar E (2021). Smart phone usage, sleep quality and depression in university students. Int J Soc Psychiatry.

[29] Yang SY, Chen MD, Huang YC, Lin CY, Chang JH (2017). Association between smartphone use and musculoskeletal discomfort in adolescent students. J Community Health.

[30] Mustafaoglu R, Yasaci Z, Zirek E, Griffiths MD, Ozdincler AR (2021). The relationship between smartphone addiction and musculoskeletal pain prevalence among young population: a cross-sectional study. Korean J Pain.

[31] Santander-Hernández FM, Peralta CI, Guevara-Morales MA, Díaz-Vélez C, Valladares-Garrido MJ (2022). Smartphone overuse, depression & anxiety in medical students during the COVID-19 pandemic. PLoS One.

[32] Kuss DJ, Griffiths MD (2011). Online social networking and addiction--a review of the psychological literature. Int J Environ Res Public Health.

[33] Cleary M, West S, Visentin D (2020). The mental health impacts of smartphone and social media use. Issues Ment Health Nurs.

[34] Huber R, Treyer V, Borbély AA (2002). Electromagnetic fields, such as those from mobile phones, alter regional cerebral blood flow and sleep and waking EEG. J Sleep Res.

[35] Mohler E, Frei P, Fröhlich J, Braun-Fahrländer C, Röösli M (2012). Exposure to radiofrequency electromagnetic fields and sleep quality: a prospective cohort study. PLoS One.

[36] Alotaibi MS, Fox M, Coman R, Ratan ZA, Hosseinzadeh H (2022). Smartphone addiction prevalence and its association on academic performance, physical health, and mental well-being among university students in Umm Al-Qura University (UQU), Saudi Arabia. Int J Environ Res Public Health.

[37] Peng A, Lin Z, Zhu C (2022). Relationship of psychiatric disorders and sleep quality to physical symptoms in coronary artery disease. J Nerv Ment Dis.

